# Chronic Osteomyelitis and Pathologic Fractures Following Radiation-Treated Sarcoma Complicated by Knee Effusion: A Case Report

**DOI:** 10.7759/cureus.107087

**Published:** 2026-04-15

**Authors:** Waseem Syed, Justin McGinnis, Stephen Westfall, Joe Bhagratie

**Affiliations:** 1 Medicine, Lake Erie College of Osteopathic Medicine, Bradenton, USA; 2 Orthopedics, Baptist Medical Center Jacksonville, Jacksonville, USA

**Keywords:** fracture, infection, osteomyelitis, osteosarcoma, radiology

## Abstract

Chronic osteomyelitis in the setting of recent soft tissue cancers, also known as sarcoma, and radiation therapy presents a clinical challenge due to poor wound healing and impaired vascularity. We present the case of a 64-year-old female patient with a history of sarcoma treated with proton beam therapy who developed chronic osteomyelitis, abscess formation, and pathologic fractures of the tibia and fibula. The course was further complicated by recurrent knee effusion and concerns for septic arthritis. Despite repeated surgical recommendations for above-knee amputation and antibiotic therapy, the patient elected to pursue conservative management. This case highlights the interplay between cancer, tissue damage from radiation therapy, and chronic infection, as well as challenges in achieving adequate treatment.

## Introduction

Primary bone and soft tissue sarcomas are rare but aggressive malignancies that account for a small proportion of adult cancers, yet carry significant morbidity and mortality due to their locally invasive nature and potential for metastasis [1]. Advances in imaging, surgical techniques, and adjuvant therapies have significantly improved survival outcomes over recent decades. As a result, there has been a major shift in treatment strategy toward limb preservation, whenever oncologically safe, with limb-sparing surgery now considered the standard of care in appropriately selected patients [2]. Successful limb salvage requires complete tumor resection with negative margins while maintaining acceptable functional outcomes, often necessitating complex reconstruction using allografts, endoprostheses, or vascularized bone grafts.

Despite these advances, limb preservation is not always feasible. Tumors involving critical neurovascular structures, extensive soft tissue compromise, or recurrent disease may necessitate amputation to achieve local control and optimize patient survival [3]. The decision between limb-sparing approaches and amputation is multifactorial, involving tumor characteristics, patient comorbidities, expected functional outcomes, and patient preferences.

Radiation therapy plays a crucial role in the management of many sarcomas, either as a neoadjuvant or adjuvant modality to improve local control [4]. Proton beam therapy, in particular, has gained increasing use due to its ability to deliver high-dose radiation to the tumor while minimizing exposure to surrounding healthy tissues. However, radiation is not without long-term consequences. Chronic radiation-induced changes include fibrosis, microvascular damage, reduced tissue elasticity, and impaired regenerative capacity [5]. These alterations predispose affected tissues to delayed healing, pathologic fractures, and increased susceptibility to infection, particularly in weight-bearing bones.

One of the most challenging complications in this setting is chronic osteomyelitis, a persistent and often refractory bone infection characterized by necrotic bone (sequestrum), biofilm formation, and limited antibiotic penetration [6]. The pathophysiology is closely linked to impaired vascular supply and compromised host immune response, both of which are exacerbated in previously irradiated tissues [7]. Patients who undergo surgical reconstruction following tumor resection are at a particularly high risk due to the presence of foreign materials, prior tissue disruption, and altered local anatomy.

Management of chronic osteomyelitis in this population is complex and typically requires a multidisciplinary approach, combining prolonged targeted antibiotic therapy with surgical intervention such as debridement, hardware removal, or reconstruction. In severe or refractory cases, especially when there is extensive bone destruction or failure of prior salvage attempts, amputation may ultimately be necessary to control infection and improve quality of life [8].

This case highlights the complex interplay between prior sarcoma treatment, radiation-induced tissue injury, and the development of chronic osteomyelitis, culminating in progressive joint destruction and functional decline. It underscores the long-term complications of multimodal cancer therapy and the importance of individualized, patient-centered decision-making in managing these challenging cases.

## Case presentation

A 64-year-old female patient with a complex medical history significant for sarcoma of the left lower extremity treated with proton beam radiation therapy in 2015 presented for evaluation of new-onset swelling of the left knee following moderate physical activity on April 16, 2025. Her oncologic course had been complicated by long-term sequelae of radiation, including poor tissue vascularity and impaired wound healing. Over the years, she developed chronic osteomyelitis involving the proximal tibia and fibula, which was further complicated by intraosseous abscess formation and multiple pathologic fractures, resulting in significant structural compromise of the affected limb.

The patient reported that the swelling began acutely after exercise and was localized primarily to the knee joint, with associated discomfort and limited range of motion. She denied systemic symptoms such as fever, chills, night sweats, or generalized fatigue, which made an acute systemic infectious process less likely at presentation. She attempted conservative symptomatic management at home, including rest, ice application, and nonsteroidal anti-inflammatory drugs, which provided only partial and temporary relief.

Her clinical course had been further complicated by the presence of a chronic, non-healing wound over the left lower extremity with intermittent purulent drainage. Prior wound cultures demonstrated polymicrobial infection, including methicillin-sensitive *Staphylococcus aureus*, *Pseudomonas aeruginosa*, and group B *Streptococcus*, reflecting both skin flora and opportunistic organisms commonly associated with chronic wounds and compromised tissue beds. She previously completed an extended eight-week course of intravenous antibiotic therapy consisting of daptomycin and cefepime, followed by transition to oral levofloxacin for suppressive therapy. However, her antibiotic regimen was complicated by gastrointestinal intolerance and a reported mild hypersensitivity reaction to cephalosporins, limiting optimal antimicrobial options.

Given the chronicity of infection, progressive bony destruction, and poor likelihood of definitive eradication of infection due to radiation-associated tissue damage, she underwent multiple evaluations by orthopedic surgery. Above-knee amputation was recommended as the most definitive treatment option to control infection, alleviate symptoms, and prevent further complications such as systemic spread or worsening structural instability. Despite thorough counseling regarding risks and benefits, the patient declined surgical intervention, expressing a preference for limb preservation and conservative management.

Her case represents a significant therapeutic challenge, highlighting the interplay between prior radiation therapy, chronic infection, impaired wound healing, and patient-centered decision-making. The current presentation of acute knee swelling raises concern for possible superimposed processes, including reactive effusion, septic arthritis, progression of osteomyelitis, or mechanical instability related to prior pathologic fractures, warranting further diagnostic evaluation.

Examination of the left knee revealed moderate joint effusion, tenderness along the medial and lateral joint lines and patella, decreased range of motion (extension to 10 degrees, flexion to 90 degrees), chronic open wound over the left lateral leg with serous drainage and no surrounding erythema, and persistent lower extremity edema.

Laboratory findings (Table 1) showed white blood cell count within the normal range, hemoglobin of 9.5 g/dL (consistent with chronic anemia), and no acute inflammatory markers available during follow-up appointment. Microbiology prior wound cultures revealed methicillin-sensitive *Staphylococcus aureus*, *Pseudomonas aeruginosa*, and group B *Streptococcus*.

**Table 1:** Laboratory and microbiology findings with reference values

Test/Parameter	Result	Reference Value	Interpretation
White blood cell count	Normal	4,000–11,000 cells/μL	Within normal range
Hemoglobin	9.5 g/dL	12–16 g/dL (female)	Consistent with chronic anemia
Acute inflammatory markers	Not available	C-reactive protein: <10 mg/L; erythrocyte sedimentation rate (ESR): <20 mm/hr	Follow-up data unavailable
Wound culture	Methicillin-sensitive *Staphylococcus aureus*	Not applicable	Pathogenic bacteria identified
Wound culture	*Pseudomonas aeruginosa*	Not applicable	Pathogenic bacteria identified
Wound culture	Group B *Streptococcus*	Not applicable	Pathogenic bacteria identified

Imaging

**Figure 1:**
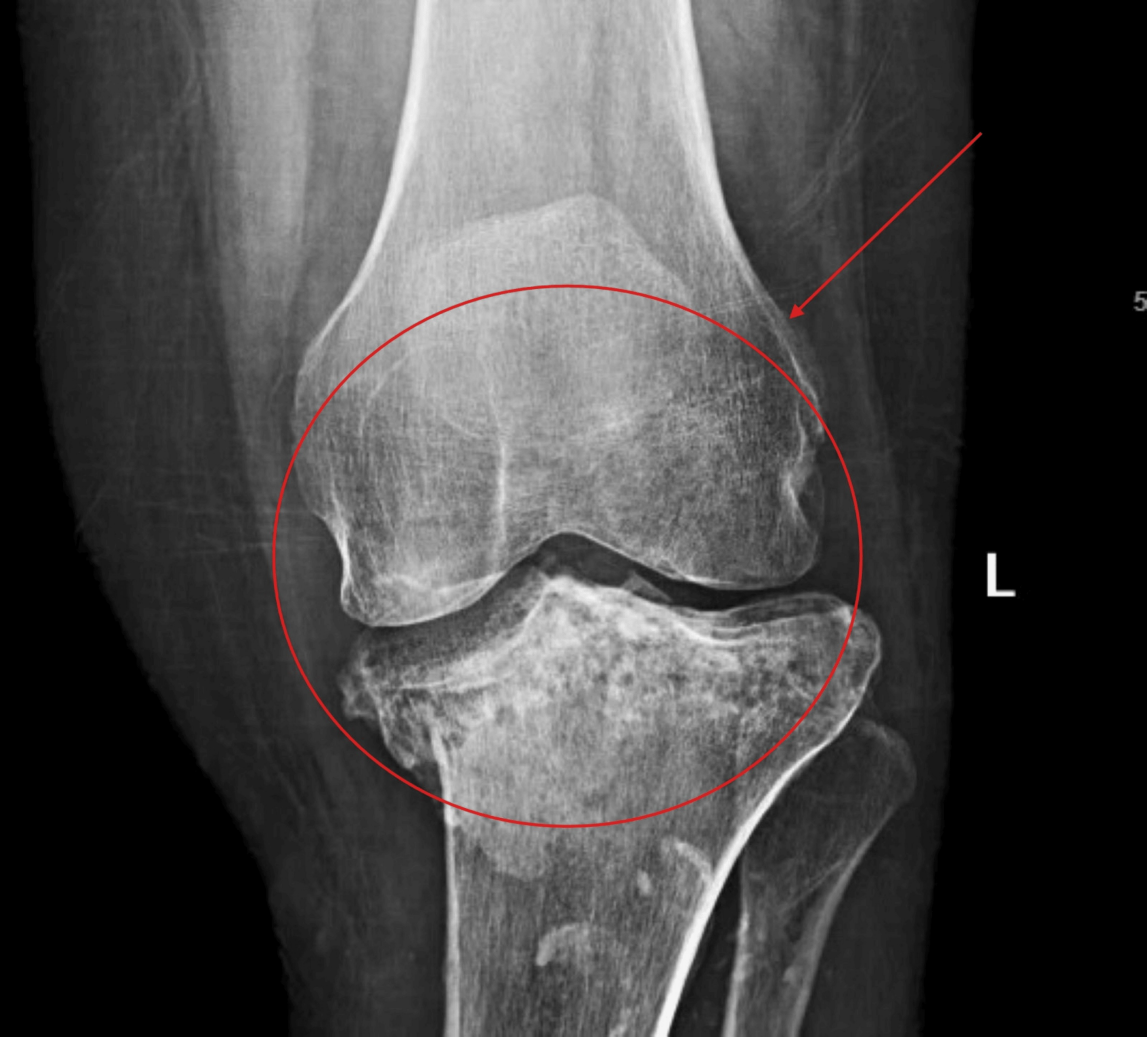
Baseline knee X-ray showing a proximal tibial lesion (2019)

**Figure 2:**
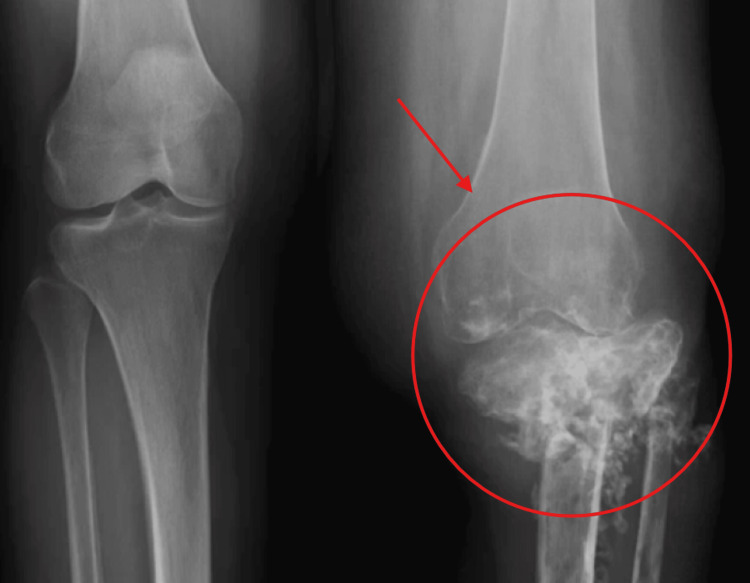
Plain radiograph of the bilateral knee and tibia/fibula (current)

**Figure 3:**
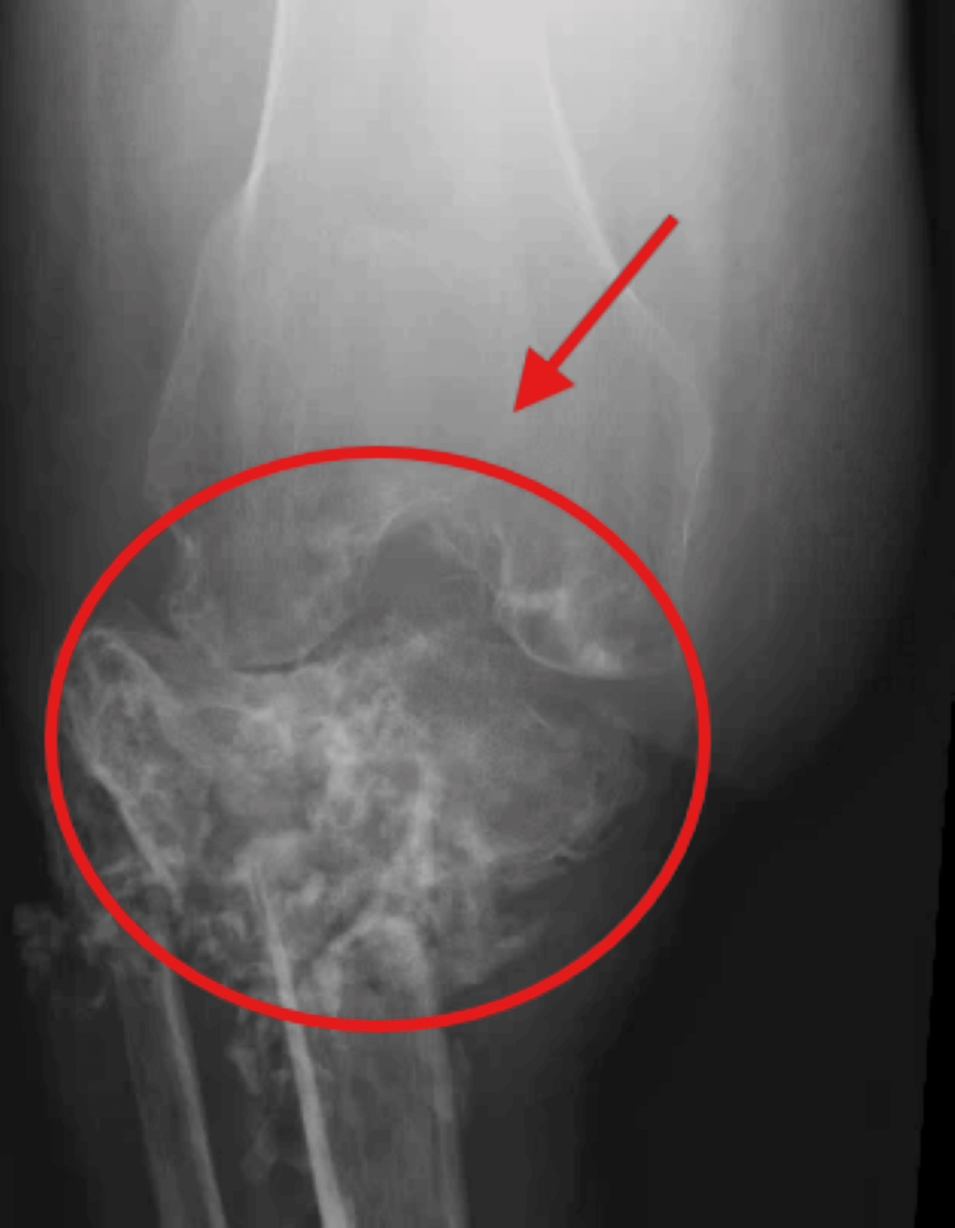
Plain radiograph (lateral view) of the left knee and tibia/fibula (current)

**Figure 4:**
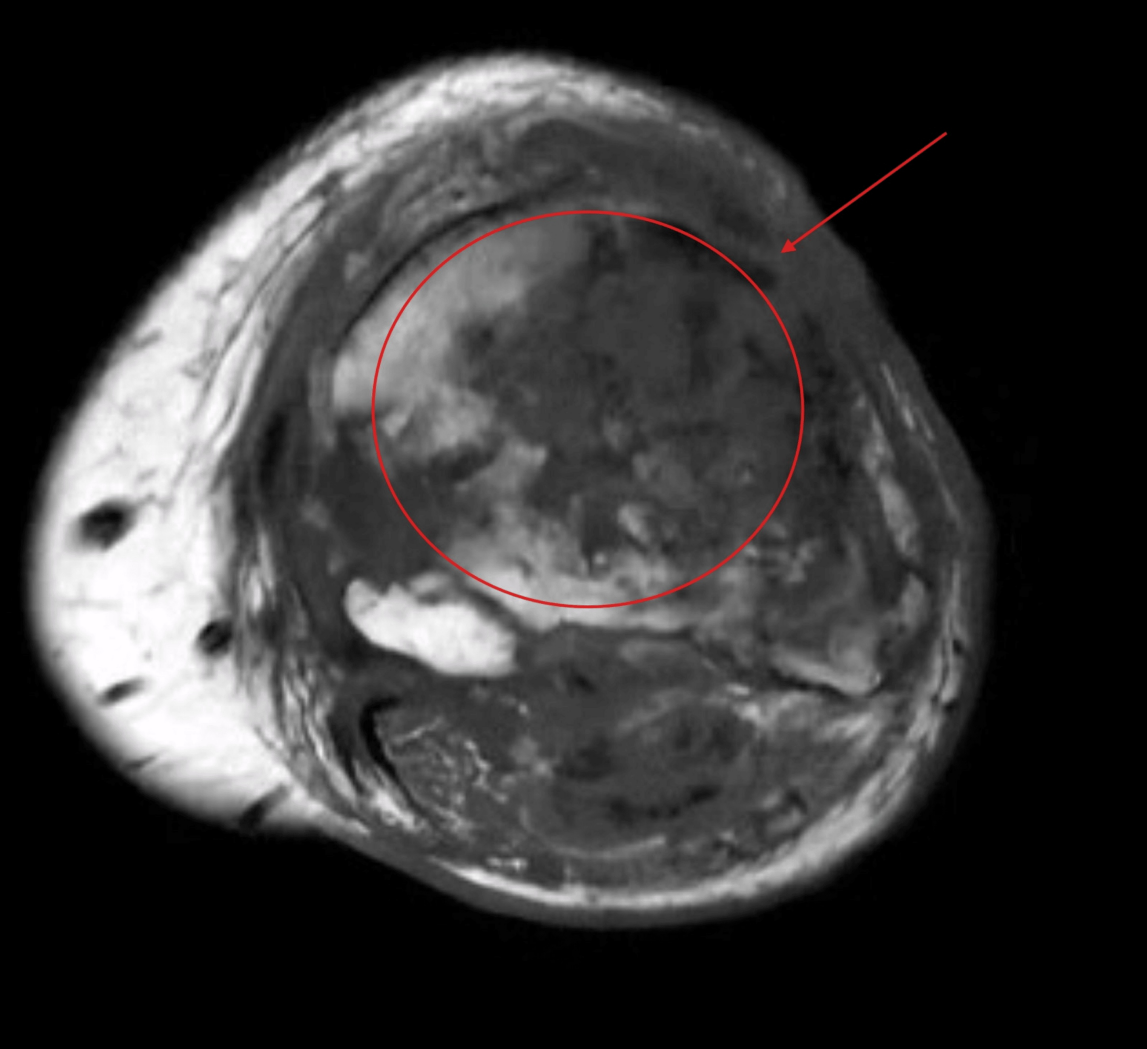
Cross-sectional MRI depicting extensive intramedullary tumor burden in osteosarcoma (current)

Figure 1 depicts the initial tibial lesions in 2019 showing infiltrative process of the tibial plateau. Figure 2 and Figure 3 show weight-bearing plain radiographs of the patient’s left knee and tibia/fibula, revealing pathologic fractures of the proximal tibia and fibula, extensive osteomyelitis and osteonecrosis, large intraosseous abscess (8.4cm), moderate joint effusion concerning for septic arthritis, and associated soft tissue infection and tendon involvement. MRI without contrast showed similar findings (Figure 4).

This patient’s presentation is consistent with chronic osteomyelitis with intraosseous abscess, radiation-induced osteonecrosis, pathologic fractures of the proximal tibia and fibula, knee effusion with concern for septic arthritis, and chronic non-healing wound.

Given the absence of systemic signs of infection and the high risk of introducing infection, joint aspiration was deferred. Management included anti-inflammatory therapy with celecoxib, local supportive care with ice, elevation, and topical diclofenac, activity modification and protected ambulation, and close outpatient monitoring. The patient continues multidisciplinary follow-up with orthopedics, infectious disease, oncology, and wound care. Despite repeated counseling regarding the benefits of surgical intervention, including above-knee amputation for definitive source control, this patient declined operative management.

## Discussion

Chronic osteomyelitis in the setting of prior sarcoma treatment and radiation therapy represents a particularly challenging clinical scenario due to the cumulative effects of tissue damage, impaired vascularity, and structural compromise. Radiation, including proton beam therapy, induces fibrosis, endarteritis, and reduced bone perfusion, predisposing irradiated tissue to osteoradionecrosis and impaired healing [5,7]. This hypovascular environment diminishes antibiotic delivery and immune cell infiltration, limiting the efficacy of systemic therapy and contributing to the persistence of chronic infections [6,9].

Surgical management of primary bone and soft tissue sarcomas, even with limb-sparing intent, often removes substantial bone and soft tissue, including periosteum, neurovascular bundles, and surrounding musculature, further impairing local tissue resilience [2]. Reconstruction using endoprostheses, allografts, or vascularized bone grafts introduces foreign material that may serve as a nidus for biofilm formation and recurrent infection, particularly in the context of impaired vascularity [2]. These combined factors - irradiated bone, prior surgery, and foreign material - create a high-risk environment for chronic osteomyelitis, as seen in this patient.

Our patient’s presentation with pathologic fractures of the proximal tibia and fibula, extensive intraosseous abscess, and knee effusion illustrates the severity of structural compromise in irradiated bone. Pathologic fractures are more likely in irradiated bone due to decreased osteoblastic activity and impaired remodeling capacity and are often associated with delayed or incomplete healing [5]. MRI proved essential in delineating the extent of osteomyelitis, intraosseous abscess, soft tissue involvement, and joint effusion, which raised concern for septic arthritis - a condition that, if untreated, can rapidly progress to joint destruction and systemic complications [10].

Management of chronic osteomyelitis in this context requires careful consideration of both medical and surgical options. While prolonged antibiotic therapy is standard, it is rarely sufficient in the presence of large abscesses, sequestra, or structural instability [6,8]. Surgical interventions, including aggressive debridement or above-knee amputation, remain the definitive means of achieving source control. Amputation, while effective for eradicating infection, carries substantial functional, psychological, and quality-of-life implications, emphasizing the importance of patient-centered decision-making and shared discussions regarding risks, benefits, and alternatives [11,12].

Non-operative management, as chosen by this patient, typically involves a combination of local wound care, activity modification, anti-inflammatory therapy, and close monitoring. Such approaches may provide symptomatic relief and slow progression, but they do not reliably prevent recurrent infection or further structural deterioration [6,7]. This underscores the limited efficacy of conservative strategies in patients with extensive osteonecrosis, abscess formation, and pathologic fractures.

Emerging adjunctive therapies, including local antibiotic delivery via beads or spacers, negative pressure wound therapy, and hyperbaric oxygen, have shown promise in select cases by improving local antibiotic concentration and enhancing tissue oxygenation and healing. However, evidence in irradiated limbs remains limited, and these interventions rarely replace the need for definitive surgical management [6,8].

This case highlights the complex interplay of prior malignancy, radiation-induced tissue damage, chronic infection, and structural compromise. It also underscores the importance of multidisciplinary care, integrating orthopedic surgery, infectious disease, oncology, and wound management, and emphasizes the critical role of individualized, patient-centered decision-making. Clinicians must balance the need for aggressive source control with patient preferences, functional outcomes, and psychosocial considerations. Ultimately, this case illustrates both the limitations of conservative management in structurally compromised irradiated limbs and the need for ongoing research into strategies that improve bone perfusion, infection control, and long-term outcomes in this high-risk population [12].

## Conclusions

Chronic osteomyelitis following sarcoma treatment and radiation therapy represents a severe and often refractory condition, driven by compromised vascularity, impaired wound healing, and structural bone damage. This case illustrates the significant limitations of conservative management in the setting of extensive bone involvement, intraosseous abscess formation, and chronic soft tissue compromise. It also highlights the complexity of clinical decision-making when standard definitive interventions, such as surgical debridement or amputation, are declined. In such scenarios, management must be individualized and patient-centered, balancing infection control, symptom management, functional preservation, and quality of life while maintaining close multidisciplinary follow-up.
